# Maternal, newborn, and child health continuum of care: completion and associated factors in Xiengkhuang and Huaphanh, Lao PDR

**DOI:** 10.1186/s41182-026-01066-7

**Published:** 2026-09-04

**Authors:** Hyejung Han, Khammany Phommachanh, Sung Hye Kim

**Affiliations:** 1 Department of Public Health Sciences, Graduate School of Hanyang University, Seoul, Korea; 2 Center for Global Health Practice, Institute of Health and Society, Hanyang University College of Medicine, Seoul, Korea; 3 National Center for Mother and Child Health, Ministry of Health, Vientiane, Lao PDR

## Abstract

**Background:**

Completing the full continuum of maternal, newborn, and child health (MNCH) care remains challenging in remote Lao PDR, which has among the highest maternal and neonatal mortality rates in Southeast Asia. Evidence on continuum of care (CoC) completion and its determinants is limited for Xiengkhuang and Huaphanh, two underserved provinces.

**Methods:**

A community-based cross-sectional household survey was conducted in February 2023 using stratified three-stage cluster sampling across eight districts. Women aged 15–49 with a live birth in the preceding 24 months were enrolled (*n* = 380). The maternal and newborn health CoC (MNH CoC) required ≥ 4 antenatal care (ANC) visits, skilled birth attendance, postnatal care for mother and newborn, and postpartum family planning (*n* = 367). An extended MNCH CoC incorporating six childhood vaccines was assessed among women with a child aged 12–23 months (*n* = 152). Province-stratified multivariable logistic regression models with cluster-robust standard errors were fitted for MNH CoC; a pooled model was used for the extended continuum.

**Results:**

Overall, 36.2% completed the MNH CoC and 22.4% completed the extended MNCH CoC. Completion was higher in Xiengkhuang than Huaphanh (MNH CoC: 49.7% vs. 22.2%; extended: 28.9% vs. 14.5%). In Xiengkhuang, Tai Folk or other religious affiliation (vs. Buddhist) was associated with lower MNH CoC completion (adjusted odds ratio [AOR] 0.10, 95% confidence interval [CI] 0.01–0.66). In Huaphanh, ethnic-minority status (AOR 0.17, 95% CI 0.03–0.96) and living 6–10 km (AOR 0.16, 95% CI 0.03–0.94) or > 10 km (AOR 0.13, 95% CI 0.02–0.83) from the nearest health facility were independently associated with lower completion. For the extended MNCH CoC, ANC initiation at ≥ 3 months was associated with lower completion (AOR 0.27, 95% CI 0.10–0.70).

**Conclusions:**

MNCH CoC completion was low and differed markedly by province, reflecting local socioeconomic and geographic disparities. Determinants varied by context, underscoring the need for province-tailored strategies. Across the extended continuum, late ANC initiation was a key barrier, highlighting first-trimester engagement as a programmatic priority. Addressing structural and socio-cultural barriers and strengthening linkage from delivery to postnatal and child health services are critical to improving continuity.

**Supplementary Information:**

The online version contains supplementary material available at https://doi.org/10.1186/s41182-026-01066-7.

## Introduction

Continuity of maternal, newborn, and child health (MNCH) care is critical for improving survival and health across the life course and remains a global health priority [1]. The continuum of care (CoC) refers to the delivery of essential interventions across key stages—pregnancy, childbirth, the postnatal period, and early childhood—so that women and children receive timely services without gaps [1, 2]. Because service contact does not necessarily reflect effective coverage or quality, assessing completion of key contacts can help identify missed opportunities while highlighting where quality gaps may remain. However, despite global progress in reducing maternal and child mortality since 2000, preventable deaths and inequities in access and continuity of MNCH services persist, particularly in low- and middle-income countries (LMICs) [3–9].

In the Lao People’s Democratic Republic (Lao PDR), MNCH indicators have improved, but disparities remain marked in remote and rural areas [10, 11]. The country continues to have among the highest maternal and neonatal mortality rates in Southeast Asia [12], with an estimated maternal mortality ratio (MMR) of 112 deaths per 100,000 live births [13] and a neonatal mortality rate (NMR) of approximately 12 deaths per 1,000 live births in 2023 [14]. In response, the government has implemented strategies to strengthen MNCH service delivery, including integrated packages of services [15]. Within this national picture, the two study provinces diverge from national averages and from each other. In the most recent Lao Social Indicator Survey, antenatal care content coverage was 72% in Xiengkhuang but only 32% in Huaphanh (national 53%), institutional delivery was 88% versus 56% (national 78%), and postnatal care for mothers within 2 days was 80% versus 46% (national 64%); childhood immunization, by contrast, was slightly higher in Huaphanh (51%) than in Xiengkhuang (40%), while under-five mortality was 37 versus 22 per 1,000 live births [16].

Xiengkhuang and Huaphanh provinces face persistent barriers related to geographic isolation, socioeconomic disadvantages, and limited health system capacity [12, 17, 18]. Since 2014, the Government of Korea, through a bilateral initiative, has partnered with Lao health authorities to strengthen the healthcare system and support MNCH service delivery in these underserved areas. During the study period, both provinces were also covered by the World Bank-supported Health and Nutrition Services Access Project (2020–2025), which used results-based financing and community engagement to strengthen maternal, newborn, child health, immunization, and nutrition services in four northern provinces, including Xiengkhuang and Huaphanh [19]. Nonetheless, evidence remains limited on CoC completion rates and factors associated with CoC completion in these provinces. Previous research in rural Khammouane province investigated MNCH service utilization using a modified composite coverage index [10], but comparable evidence is needed for Xiengkhuang and Huaphanh to identify context-specific barriers and enablers. Evidence on CoC completion in the region reflects both framings used here. Studies applying the standard maternal and newborn CoC report low completion and consistent drop-off at the postnatal stage; a recent national analysis in Lao PDR using an ANC4+, skilled birth attendance, and postnatal-care definition found completion of only 3.3%, with postnatal care the principal point of attrition and Hmong–Mien women least likely to complete [20]. Studies applying an extended MNCH framework that adds childhood immunization report similarly low completion; a population-based analysis of lower-middle-income countries in Southeast Asia likewise documented low completion of the extended continuum, broadly consistent with the level observed in the present study [21].

This study assessed completion of the MNCH CoC in Xiengkhuang and Huaphanh and examined factors associated with completion. We operationalized a maternal and newborn health continuum of care (MNH CoC) using recommended contacts across pregnancy, delivery, and the postnatal period, and further assessed an extended MNCH CoC that incorporated childhood vaccination to capture continuity from pregnancy through early childhood.

## Materials and methods

### Study area and period

**Fig. 1:**
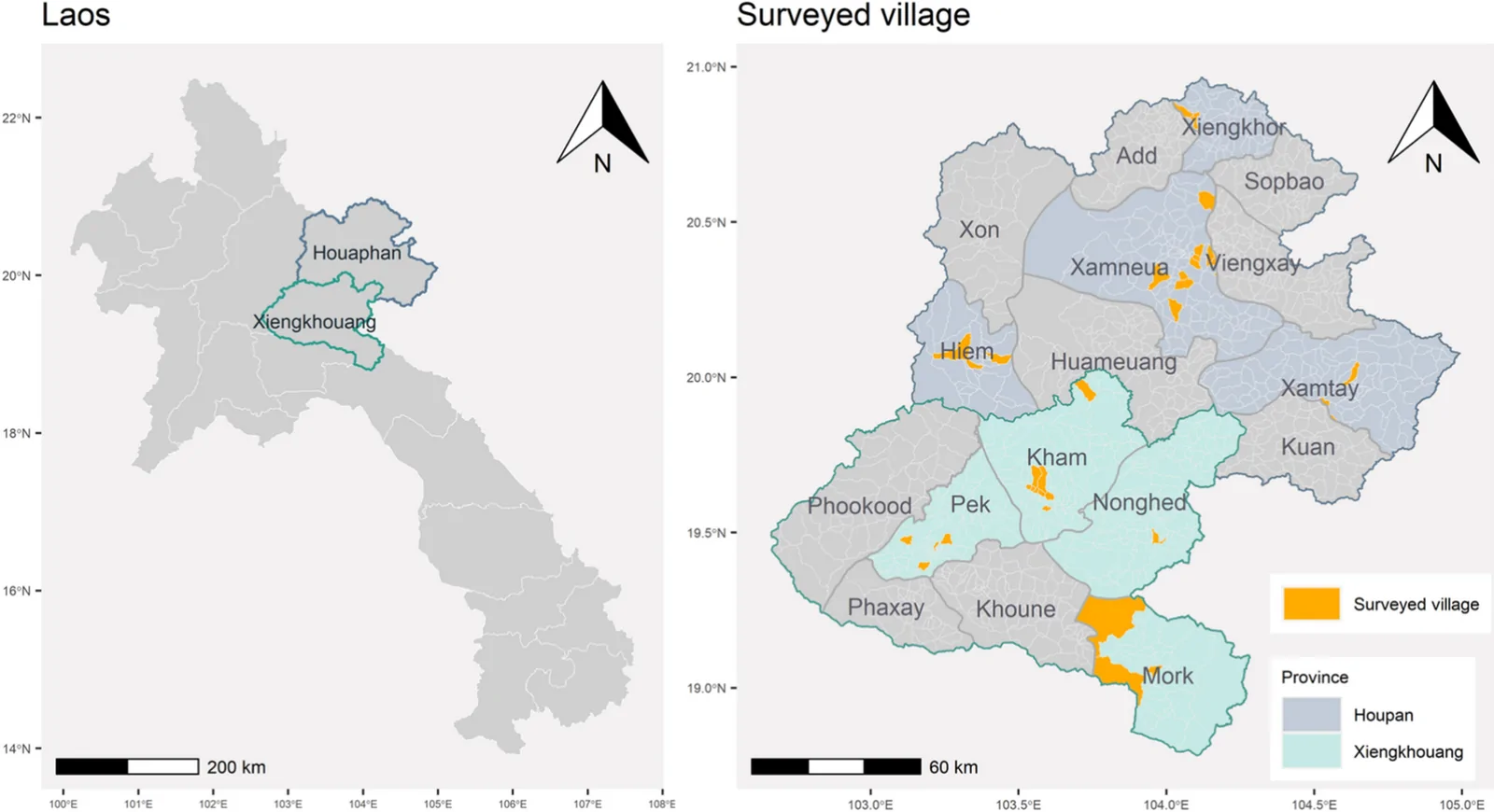
Map of the study location and surveyed villages

This study was conducted in Xiengkhuang and Huaphanh provinces, located in the central and northern regions of the Lao PDR. It involved four type A districts from each province, specifically those with available comprehensive emergency obstetric and newborn care services at the time of the survey (Fig. 1). Data collection was carried out between 12 and 27 February 2023. This 2-week period refers to the field data-collection phase only. Data collection was carried out concurrently by multiple trained interview teams working in parallel across the selected districts, following prior household listing and village preparation; the fixed quota of 10 women per village (190 per province) was fully achieved within this window, and the period was sufficient to complete the planned sampling and interviews. In the Lao health system, services are organized across central, provincial, district, and health-center levels, with district hospitals classified by the scope of services they provide. Type A district hospitals are the most fully equipped at the district level and are designated to provide comprehensive emergency obstetric and newborn care (CEmONC), including cesarean section and blood transfusion, whereas lower-tier district hospitals and health centers typically offer only basic emergency obstetric and newborn care [22]. The four study districts in each province were, therefore, among the better-resourced settings for maternal and newborn care within their respective provinces.

### Study design

This study employed a community-based cross-sectional study design.

### Study population

The study participants comprised women of reproductive age (15–49 years) residing in the sampled households in Xiengkhuang and Huaphanh provinces who had delivered a live birth within the preceding 24 months. One eligible woman per household was randomly chosen to participate. For the extended MNCH continuum analysis, we assessed a subsample of women whose youngest child was aged 12–23 months at the time of interview for whom immunization information was collected from home-based cards, where available; otherwise, maternal recall was used.

### Sample size and sampling procedure

**Fig. 2:**
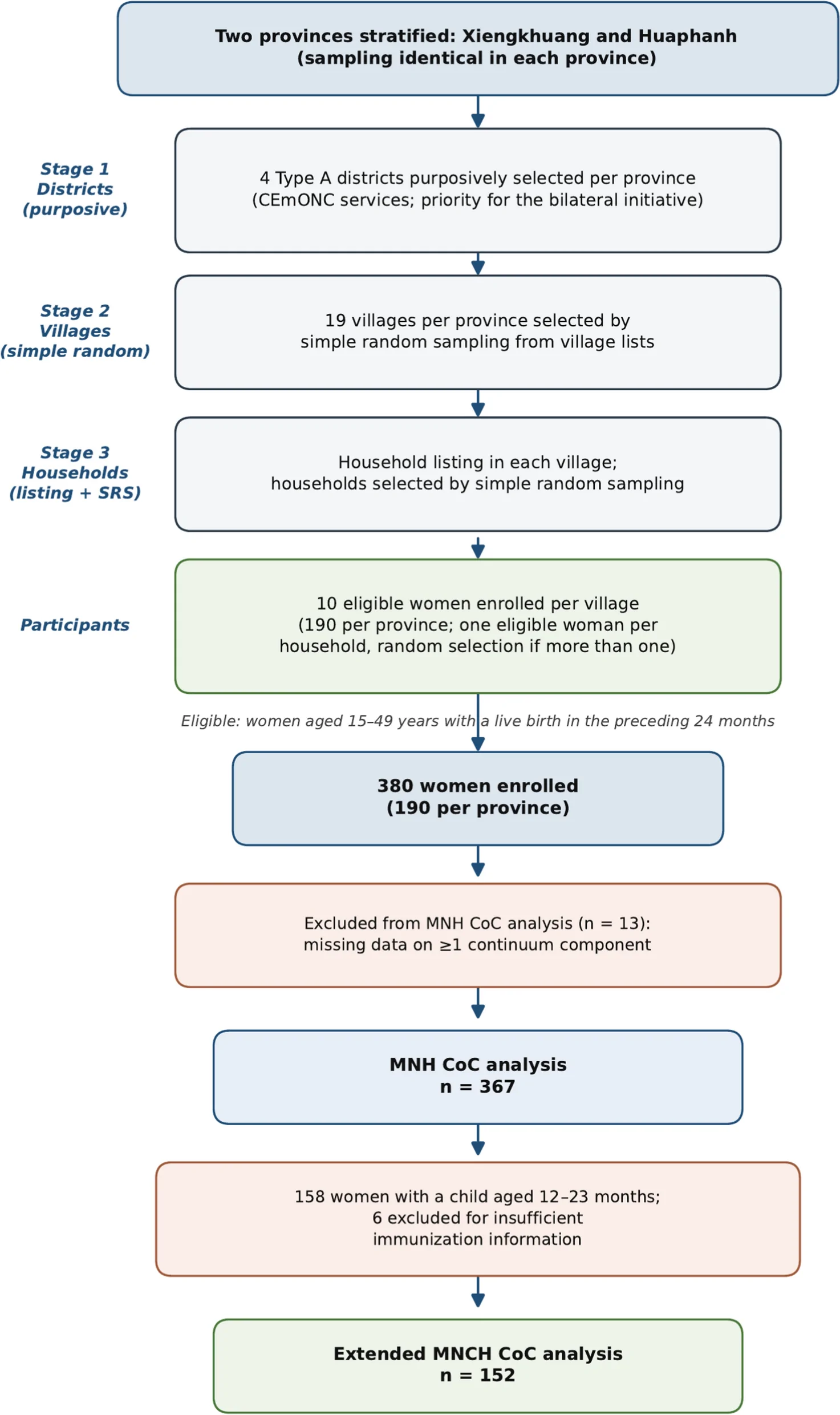
Flow diagram of the sampling and participant selection process

We conducted a three-stage cluster household survey, stratified by province. In stage 1, four districts per province were purposively selected, because they had a Type A district hospital with comprehensive emergency obstetric and newborn care (CEmONC) services available at the time of the survey and were priority areas for the bilateral health initiative. In stage 2, 19 villages per province were selected using simple random sampling from official village lists provided by district authorities. In stage 3, within each selected village, households were selected using household listing and simple random sampling, and eligible women were enrolled until 10 participants per village were interviewed (190 per province; 380 total). If more than one eligible woman was present in a household, one was selected by simple random selection. This aggregate sample of 190 women per province provides a statistical precision (margin of error) of approximately ± 7–10% at a 95% confidence interval for point coverage estimates, based on a design effect of 2 and an expected proportion of ~ 50% (most conservative estimate).The multi-stage selection of districts, villages, households, and participants is summarized in a sampling flow diagram (Fig. 2).

### Data collection tool and procedure

Data collection utilized a semi-structured questionnaire, which was adapted from the generic lot quality assurance sampling (LQAS) household questionnaire [23] to fit the Lao context. The modification was done in collaboration with Health Officers overseeing MCH programs at both the Provincial and District Health Offices. Notable changes included adjusting sociodemographic items to reflect local ethnic and religious diversity, incorporating questions aligned with Lao PDR’s national immunization schedule and maternal health service usage, and adding geographic and accessibility details (such as road conditions and distances). The questionnaires were initially developed in English, translated to Lao for better comprehension by participants, and then back-translated into English to ensure accuracy. Each eligible woman was interviewed separately by trained health officers. Antenatal care attendance and skilled birth attendance were ascertained primarily from maternal recall during the interview, supplemented by home-based records (antenatal care and child health cards) when these were available at the time of interview. A single common questionnaire was used across all districts in both provinces; the context-specific adaptations concerned only the response options for locally relevant sociodemographic items (for example, ethnic and religious categories) and did not alter the wording, sequence, or measurement of the maternal, newborn, and child health service indicators, so that these core measures remained identical and directly comparable across all study sites. Interviewer training and standardized field procedures were shared across teams to further ensure consistency.

### Study variables

#### Dependent variables

**Fig. 3:**
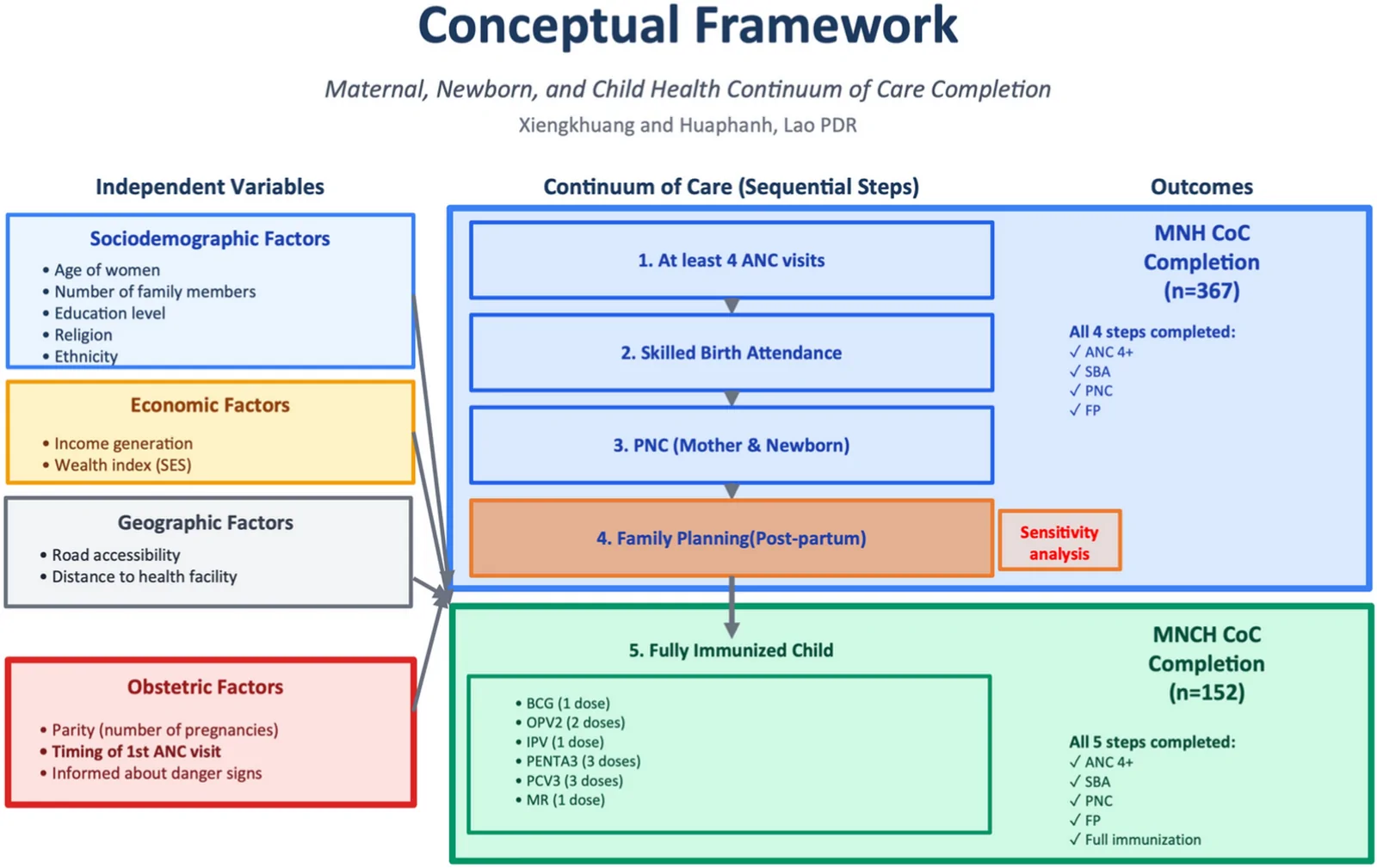
Conceptual framework of factors associated with MNCH continuum-of-care completion

Continuum completion was defined using an all-or-nothing binary indicator: women were classified as completing the continuum only if all required contacts in the definition were met [10, 24–29]. A woman was considered to have completed the MNH CoC if she reported: (1) at least four antenatal care visits (ANC4+); (2) skilled birth attendance (SBA), defined as delivery assisted by a doctor, nurse, or midwife; (3) postnatal care for the mother (PNCM) and newborn (PNCN) provided by a skilled provider within 6 weeks after delivery, either at a facility or at home; and (4) postpartum family planning (FP) counseling or services provided by a health professional within 6-week postpartum. We retained an ANC4+ threshold rather than the WHO 2016 recommendation of at least eight antenatal contacts, because ANC4+ was the indicator specified in the Lao national maternal health guidelines in use at the time of the survey, is the measure routinely recorded in Lao health information systems and national surveys, and is used in most comparable continuum-of-care studies in the region, supporting cross-study comparability; eight-contact coverage also remains very low in this setting, which would leave few women meeting the threshold [30, 31]. Variable selection was guided by a conceptual framework (Fig. 3) adapted from established frameworks for MNCH service utilization, which categorizes potential determinants as enabling, predisposing, and need-related factors [32, 33]. This approach provides a clear, policy-relevant threshold for identifying individuals who miss any recommended contact and, therefore, experience a break in continuity.

For women with a child aged 12–23 months, we additionally assessed an extended MNCH CoC incorporating six childhood vaccines: one dose of Bacillus Calmette–Guérin (BCG), two doses of oral polio vaccine (OPV2), one dose of inactivated polio vaccine (IPV), three doses of the pentavalent vaccine (PENTA3), three doses of pneumococcal conjugate vaccine (PCV3), and one dose of the measles–rubella (MR) vaccine. Vaccination status was ascertained from home-based immunization cards when available; when cards were not available, the mother’s recall was used. Because OPV3 was not consistently captured in the survey records, we used OPV2 as a proxy measure for receipt of oral polio vaccination in the extended continuum; this deviation from the standard definition is addressed as a limitation.

#### Independent variables

Independent variables in this study included sociodemographic, economic, and obstetric factors (Fig. 3). Socio-demographic and economic variables considered were age of the women, number of family members, education level, income generation, religion, ethnicity, road accessibility, distance to the nearest health facility, and wealth index. Obstetric factors included parity (number of pregnancies) and timing of the first ANC visit. This study also looked at whether participants were informed about danger signs during pregnancy. Income generation was assessed as a binary indicator of whether the woman engaged in any income-earning activity (yes/no), rather than regular employment or a measure of stable household income.

### Data quality control

To ensure data quality, several steps were implemented throughout the study process. Pre-trained health officers conducted the interviews, and the questionnaires were translated into Lao and then back-translated into English to check for consistency. Data entry was performed using Microsoft Excel 2021, with cleaning and editing to address errors or omissions. Each day, completed surveys were reviewed to identify and amend missing or implausible responses. Data quality was further checked by randomly comparing original paper records against the entered data.

### Data analysis

All statistical analyses were performed using STATA version 17 (StataCorp, Texas, USA). Descriptive statistics, including frequencies and percentages, summarized participant characteristics and coverage of individual services. Group differences were assessed using chi-square tests. Household socioeconomic status (SES) was derived using principal component analysis (PCA) of household assets and housing characteristics [34], with a Kaiser–Meyer–Olkin (KMO) score of at least 0.6 to confirm sampling adequacy. The wealth index was divided into quintiles, creating five SES categories: poorest (lowest 20%), poorer (20–40%), middle (40–60%), richer (60–80%), and richest (highest 20%).

To manage missing data, we applied different strategies for our two primary outcomes. For the MNH CoC, we utilized a complete-case analysis, which included 367 women after excluding 13 participants who had missing data on one or more CoC components. Conversely, for the pooled extended MNCH CoC analysis (*n* = 152), the sample was restricted to complete cases for the outcome variable, but missing covariate data (specifically the timing of the first ANC visit) were addressed using multiple imputation (MI) to preserve statistical power. We generated 20 imputed datasets using multinomial logistic regression.

We fitted bivariate logistic regression models and included variables with *p* value < 0.20 in multivariate analysis. Adjusted odds ratios (AORs) with 95% confidence intervals (CI) were reported. For the MNH CoC, multivariable models were stratified by province. For the extended MNCH CoC, a pooled multivariate model was fitted. To account for the clustered sampling design, we used village-level cluster-robust standard errors in all regression models.

We assessed multicollinearity using the variance inflation factor (VIF) (S1 Table). Model calibration was evaluated using the Hosmer–Lemeshow goodness-of-fit test (10 groups) [35]. Model explanatory power was summarized using McFadden’s pseudo-*R*^2^. Finally, sensitivity analyses were conducted to evaluate the robustness of the findings, including a results from a different imputation approach (S2 Table) and an alternative MNH CoC definition (S3 Table).

### Ethical approval and consent to participate

Ethical approval for this study was obtained from the Research Ethics Committee of the University of Health Sciences, Lao PDR (approval no. 439). Prior to survey participation, all participants provided written informed consent. Participation was entirely voluntary, and participants were informed that they could withdraw their consent at any time without any consequences. Throughout data collection, the confidentiality of all participant data was maintained. Participants aged 15–17 years were enrolled in accordance with the approved ethics protocol: informed consent was obtained from a parent or guardian, together with the assent of the adolescent participant, before the interview.

## Results

### Socio-demographic and maternal characteristics of study participants

Initially, 380 women were enrolled. After data cleaning, complete outcome data for MNH CoC were available for 367 women. The 13 women excluded had missing data on one or more CoC component. A subsample of 158 participants had a child aged 12–23 months; for these children, vaccination information was obtained from immunization cards when available and otherwise from the mother’s recall. Among the 158, we had sufficient vaccination information to determine completion of the extended MNCH continuum of care for 152 children, who were included in the extended MNCH CoC analysis.

Table 1 summarizes background characteristics of the 367 participants. Provincial differences were observed: compared with Huaphanh, participants from Xiengkhuang generally had higher educational attainment, better road accessibility, and shorter distance to health facilities. Differences were also observed in age distribution and household wealth.

**Table 1:** Socio-demographic characteristics of study participants (*n* = 367)

Variables	Number (%)			*p*-value^a^
	Total	Xiengkhuang (*n* = 187)	Huaphanh (*n* = 180)	
Age of women (years), mean [SD]	25.9 [5.7]	27.2 [5.9]	24.6 [5.1]	
15–24 years	173 (47.1)	71 (38.0)	102 (56.7)	0.001
25–34 years	164 (44.7)	96 (51.3)	68 (37.8)	
35–49 years	30 (8.2)	20 (10.7)	10 (5.6)	
Number of family members				
≤ 3	14 (3.8)	6 (3.2)	8 (4.4)	0.750
4–6	191 (52.1)	100 (53.5)	91 (50.6)	
≥ 7	162 (44.1)	81 (43.3)	81 (45.0)	
Education				
No education	27 (7.4)	4 (2.1)	23 (12.8)	< 0.001
Preschool and primary	80 (21.8)	36 (19.3)	44 (24.4)	
Middle school	100 (27.3)	49 (26.2)	51 (28.3)	
Higher	160 (43.6)	98 (52.4)	62 (34.5)	
Income generation				
Yes	260 (70.8)	118 (63.1)	142 (78.9)	0.001
No	107 (29.2)	69 (36.9)	38 (21.1)	
Religion				
Buddhism	153 (41.7)	107 (57.2)	46 (25.6)	< 0.001
Tai Folk	212 (57.8)	78 (41.7)	134 (74.4)	
Christianity	2 (0.5)	2 (1.1)	0 (0.0)	
Ethnicity				
Lao	169 (46.1)	111 (59.4)	58 (32.2)	< 0.001
Minority	198 (53.9)	76 (40.6)	122 (67.8)	
Road accessibility				
Yes	185 (50.4)	105 (56.2)	80 (44.4)	< 0.001
Partially yes	78 (21.3)	46 (24.6)	32 (17.8)	
No	104 (28.3)	36 (19.2)	68 (37.8)	
Distance to the nearest facility (km)				
≤ 5	77 (21.0)	59 (31.5)	18 (10.0)	< 0.001
6–10	135 (36.8)	68 (36.4)	67 (37.2)	
≥ 11	155 (42.2)	60 (32.1)	95 (52.8)	
Wealth index (*n* = 359)				
Poorest	71 (19.8)	22 (11.8)	49 (28.5)	< 0.001
Poorer	73 (20.3)	29 (15.5)	44 (25.6)	
Middle	72 (20.1)	33 (17.6)	39 (22.7)	
Richer	71 (19.8)	42 (22.5)	29 (16.8)	
Richest	72 (20.1)	61 (32.6)	11 (6.4)	

SD: standard deviation, km: kilometer, ^a^ *p* values from chi-square test or Fisher’s exact test when expected cell counts were < 5

### Coverage of continuum of maternal, newborn, and child health care services

The coverage of individual MNCH services differed between provinces and declined across the care sequence from pregnancy through the postnatal period and early childhood (Table 2). Coverage was highest for earlier maternal services, including skilled birth attendance (87.7%) and at least four antenatal care visits (78.8%). Coverage decreased during the postnatal period: postnatal care for mothers was 82.6%, whereas postnatal care for newborns was 61.0%, a difference of 21.6 percentage points. Uptake of postpartum family planning counseling or services within 6-week postpartum was lowest among maternal services, at 57.2% (Table 2).

**Table 2:** Maternal and newborn health service utilization (*n* = 367) and childhood immunization coverage among children aged 12–23 months (*n* = 152), by province

Variables	Category	Number (%)			*p* value^a^
		Total	Xiengkhuang (*n* = 187)	Huaphanh (*n* = 180)	
Maternal indicators (*n* = 367)					
No. of pregnancies	1	106 (28.9)	65 (34.7)	41 (22.8)	0.009
	2	111 (30.2)	45 (24.1)	66 (36.7)	
	≥ 3	150 (40.9)	77 (41.2)	73 (40.5)	
Number of antenatal care visits	No visit	13 (3.5)	0 (0.0)	13 (7.2)	< 0.001
	1–3 times	65 (17.7)	24 (12.8)	41 (22.8)	
	4–7 times	219 (59.7)	132 (70.6)	87 (48.3)	
	≥ 8 times	70 (19.1)	31 (16.6)	39 (21.7)	
Timing of 1st antenatal care visit	< 3 months	130 (35.4)	77 (41.2)	53 (29.4)	< 0.001
	≥ 3 months	224 (61.0)	110 (58.8)	114 (63.3)	
	Do not know	13 (3.5)	0 (0.0)	13 (7.2)	
Informed about danger signs during pregnancy	Yes	325 (88.3)	180 (96.3)	145 (80.5)	< 0.001
	No	30 (8.2)	7 (3.7)	23 (12.8)	
	Do not know	12 (3.3)	0 (0.0)	12 (6.7)	
Skilled birth attendance	Yes	322 (87.7)	177 (94.6)	145 (80.6)	< 0.001
	No	45 (12.3)	10 (5.4)	35 (19.4)	
Postnatal care for mother	Yes	303 (82.6)	173 (92.5)	130 (72.2)	< 0.001
	No	64 (17.4)	14 (7.5)	50 (27.8)	
Postnatal care for newborn	Yes	224 (61.0)	153 (81.8)	71 (39.4)	< 0.001
	No	143 (39.0)	34 (18.2)	109 (60.6)	
Family planning as part of PNC	Yes	210 (57.2)	120 (64.2)	90 (50.0)	0.006
	No	157 (42.8)	67 (35.8)	90 (50.0)	
Child immunization total (12–23 months, *n* = 152)			Xiengkhuang (*n* = 83)	Huaphanh (*n* = 69)	
BCG	Yes	131 (86.2)	74 (89.2)	57 (82.6)	0.244
	No	21 (13.8)	9 (10.8)	12 (17.4)	
OPV2	Yes	106 (69.7)	59 (71.1)	47 (68.1)	0.692
	No	46 (30.3)	24 (28.9)	22 (31.9)	
IPV	Yes	95 (62.5)	47 (56.6)	48 (69.6)	0.101
	No	57 (37.5)	36 (43.4)	21 (30.4)	
PENTA3	Yes	105 (69.1)	57 (68.7)	48 (69.6)	0.906
	No	47 (30.9)	26 (31.3)	21 (30.4)	
PCV3	Yes	101 (66.5)	54 (65.1)	47 (68.1)	0.691
	No	51 (33.5)	29 (34.9)	22 (31.9)	
MR	Yes	105 (69.1)	58 (69.9)	47 (68.1)	0.815
	No	47 (30.9)	25 (30.1)	22 (31.9)	

BCG: one dose of Bacillus Calmette–Guérin; OPV2: two doses of oral polio vaccine; IPV: one dose of inactivated polio vaccine; PENTA3: three doses of pentavalent vaccine; PCV3: three doses of pneumococcal conjugate vaccine; MR: one dose of measles–rubella vaccine

^a^*p* values from chi-square test or Fisher’s exact test when expected cell counts were < 5

Provincial stratification showed consistently higher coverage in Xiengkhuang than in Huaphanh across the continuum (Table 2). MNH CoC completion was 49.7% in Xiengkhuang compared with 22.2% in Huaphanh (Table 3). When the continuum was extended to include childhood vaccinations (extended MNCH CoC), completion decreased to 28.9% in Xiengkhuang and 14.5% in Huaphanh (Table 3).

**Table 3:** Coverage of MNH CoC (*n* = 367) and extended MNCH CoC including childhood immunization (*n* = 152; children aged 12–23 months) in the study sites

A) MNH CoC (*n* = 367)					
Outcome	Category	Number (%)			
		Total (*n* = 367)	Xiengkhuang (*n* = 187)	Huaphanh (*n* = 180)	*p* value^a^
MNH CoC	Yes	133 (36.2)	93 (49.7)	40 (22.2)	< 0.001
	No	234 (63.8)	94 (50.3)	140 (77.8)	

B) Extended MNCH CoC (*n* = 152; children aged 12–23 months)					
Outcome	Category	Total (*n* = 152)	Xiengkhuang (*n* = 83)	Huaphanh (*n* = 69)	*p* value^a^
MNCH CoC	Yes	34 (22.4)	24 (28.9)	10 (14.5)	0.034
	No	118 (77.6)	59 (71.1)	59 (85.5)	

MNH CoC: Continuum of Maternal and Newborn Health Care; MNCH CoC: Continuum of Maternal, Newborn and Child Health Care. Extended MNCH CoC includes childhood vaccination

^a^*p* values from chi-square test or Fisher’s exact test where appropriate

The sequential completion rates across the continuum are illustrated in S1 Fig., which shows the greatest attrition occurring at the postnatal care for the newborn and postpartum family planning steps, and again at the transition to childhood immunization.

### Factors associated with the completion of the MNH CoC

Tables 4 and 5 present bivariate and multivariate logistic regressions results for factors associated with MNH CoC completion, stratified by province. The pattern of associations differed between Xiengkhuang and Huaphanh. The province-stratified primary MNH CoC models showed adequate calibration (Hosmer–Lemeshow *p* > 0.05), with pseudo-*R*^2^ of 0.106 for Xiengkhuang (Hosmer–Lemeshow χ^2^(8) = 5.50, *p* = 0.704) and 0.252 for Huaphanh (χ^2^(8) = 5.62, *p* = 0.690).

**Table 4:** Factors associated with completing continuum of maternal and newborn care in the study sites in Xiengkhuang

Variables	Complete continuum of care (*n* = 187)		COR (95% CI)		AOR (95% CI)	
	Yes (%)	No (%)	Xiengkhuang	*p*	Xiengkhuang	*p*
Age of women (years)						
15–24 years	29 (40.9)	42 (59.2)	1		1	
25–34 years	56 (58.3)	40 (41.7)	2.03 (1.09–3.79)	0.027	1.64 (0.84–3.20)	0.147
35–49 years	8 (40.0)	12 (60.0)	0.97 (0.35–2.66)	0.946	0.75 (0.22–2.57)	0.641
Number of family members						
≤ 3	2 (33.3)	4 (66.7)	1		–	
4–6	57 (57.0)	43 (43.0)	2.65 (0.46–15.15)	0.274	–	
≥ 7	34 (42.0)	47 (58.0)	1.45 (0.25–8.40)	0.681	–	
Education						
No education	1 (25.0)	3 (75.0)	1		–	
Preschool and primary	13 (36.1)	23 (63.9)	1.70 (0.16–18.13)	0.662	–	
Middle school	25 (51.0)	24 (49.0)	3.12 (0.30–32.37)	0.339	–	
High school and above	54 (55.1)	44 (44.9)	3.68 (0.37–36.87)	0.268	–	
Income generation						
Yes	67 (56.8)	51 (43.2)	1		1	
No	26 (37.7)	43 (62.3)	0.46 (0.25–0.85)	0.013	0.64 (0.33–1.26)	0.194
Religion						
Buddhism	64 (59.8)	43 (40.2)	1		1	
Tai Folk or others	29 (36.3)	51 (63.7)	0.38 (0.21–0.70)	0.002	0.10 (0.01–0.66)	0.017
Ethnicity						
Lao	63 (56.8)	48 (43.2)	1		1	
Minority	30 (39.5)	46 (60.5)	0.50 (0.27–0.90)	0.021	6.79 (0.98–47.32)	0.053
Road accessibility						
Yes	58 (55.2)	47 (44.8)	1		1	
Partially yes	25 (54.4)	21 (45.6)	0.96 (0.48–1.94)	0.920	1.08 (0.49–2.39)	0.842
No	10 (27.8)	26 (72.2)	0.31 (0.14–0.71)	0.006	0.40 (0.16–1.01)	0.053
Distance from the nearest health facility						
< 5 km	28 (47.5)	31 (52.5)	1			
6–10 km	37 (54.4)	31 (45.6)	1.32 (0.66–2.66)	0.436	–	
> 10 km	28 (46.7)	32 (53.3)	0.97 (0.47–1.99)	0.931	–	
No. of pregnancies						
1	32 (49.2)	33 (50.8)	1		–	
2	25 (55.6)	20 (44.4)	1.29 (0.60–2.77)	0.515	–	
≥ 3	36 (46.8)	41 (53.2)	0.91 (0.47–1.76)	0.769	–	
Timing of 1st ANC visit						
< 3 months	46 (59.7)	31 (40.3)	1		1	
≥ 3 months	47 (42.7)	63 (57.3)	0.50 (0.28–0.91)	0.023	0.65 (0.34–1.26)	0.200
Informed about danger signs during pregnancy						
Yes	89 (49.4)	91 (50.6)	1		–	
No	4 (57.1)	3 (42.9)	1.36 (0.30–6.29)	0.691	–	
Wealth index						
Poorest	10 (45.5)	12 (54.5)	1		–	
Poorer	7 (24.1)	22 (75.9)	0.38 (0.12–1.27)	0.115	–	
Middle	18 (54.6)	15 (45.4)	1.44 (0.49–4.27)	0.511	–	
Richer	21 (50.0)	21 (50.0)	1.20 (0.43–3.39)	0.730	–	
Richest	37 (60.7)	24 (39.3)	1.85 (0.69–4.96)	0.222	–	

ANC: antenatal care; AOR: adjusted odds ratio; CI: confidence interval; COR: crude odds ratio; km: kilometer; OR: odds ratio. Sample sizes vary due to missing responses; denominators are shown where applicable. – indicates variable not included in the multivariable model based on the pre-specified selection criterion (*p* < 0.20 in bivariate analysis) or not estimable due to sparse cells

**Table 5:** Factors associated with completing continuum of maternal and newborn care in the study sites in Huaphanh

Variables	Complete continuum of care (*n* = 180)		COR (95% CI)		AOR (95% CI)	
	Yes (%)	No (%)	Huaphanh	*p*	Huaphanh	*p*
Age of women (years)						
15–24 years	18 (17.6)	84 (83.4)	1		–	
25–34 years	19 (27.9)	49 (72.1)	1.81 (0.87–3.78)	0.115	–	
35–49 years	3 (30.0)	7 (70.0)	2.00 (0.47–8.52)	0.349	–	
Number of family members						
≤ 3	2 (25.0)	6 (75.0)	1		–	
4–6	25 (27.5)	66 (72.5)	1.14 (0.21–6.04)	0.881	–	
≥ 7	13 (16.1)	68 (83.9)	0.57 (0.10–3.18)	0.524	–	
Education						
None, Preschool and primary	8 (11.9)	59 (88.1)	1		1	
Middle school	12 (23.5)	39 (76.5)	2.27(0.85–6.07)	0.103	2.00 (0.47–8.64)	0.351
High school and above	20 (32.3)	42 (67.7)	3.51(1.41–8.75)	0.007	1.30(0.29–5.84)	0.735
Income generation						
Yes	32 (22.5)	110 (77.5)	1		–	
No	8 (21.1)	30 (78.9)	0.92 (0.38–2.20)	0.846	–	
Religion						
Buddhism	20 (43.5)	26 (56.5)	1		1	
Tai Folk or others	20 (14.9)	114 (85.1)	0.23 (0.11–0.48)	< 0.001	0.33 (0.07–1.59)	0.169
Ethnicity						
Lao	23 (39.7)	35 (60.3)	1		1	
Minority	17 (13.9)	105 (86.1)	0.25 (0.12–0.51)	< 0.001	0.17 (0.03–0.96)	0.045
Road accessibility						
Yes	20 (25.0)	60 (75.0)	1		1	
Partially yes	15 (46.9)	17 (53.1)	2.65 (1.12–6.27)	0.027	0.75 (0.20–2.83)	0.670
No	5 (7.4)	63 (92.6)	0.24 (0.08–0.68)	0.007	1.62 (0.34–7.59)	0.542
Distance from the nearest health facility						
< 5 km	8 (44.4)	10 (55.6)	1		1	
6–10 km	19 (28.4)	48 (71.6)	0.49 (0.17–1.45)	0.199	0.16 (0.03–0.94)	0.042
> 10 km	13 (13.7)	82 (86.3)	0.20 (0.07–0.60)	0.004	0.13 (0.02–0.83)	0.032
No. of pregnancies						
1	8 (19.5)	33 (80.5)	1		–	
2	19 (28.8)	47 (71.2)	1.67 (0.65–4.27)	0.287	–	
≥ 3	13 (17.8)	60 (82.2)	0.89 (0.34–2.38)	0.822	–	
Timing of 1st ANC visit (*n* = 164)						
< 3 months	14 (29.8)	33 (70.2)	1		1	
≥ 3 months	16 (13.7)	101 (86.3)	0.37 (0.16–0.85)	0.019	0.42 (0.16–1.13)	0.085
Informed about danger signs during pregnancy						
Yes	37 (25.5)	108 (74.5)	1		–	
No	3 (8.6)	32 (91.4)	0.27 (0.08–0.95)	0.041	–	
Wealth index	(*n* = 172)					
Poorest	6 (12.2)	43 (87.8)	1		1	
Poorer	14 (31.8)	30 (68.2)	3.34 (1.15–9.72)	0.027	2.77 (0.50–15.34)	0.243
Middle	9 (23.1)	30 (76.9)	2.15 (0.69–6.70)	0.187	1.13 (0.18–7.13)	0.898
Richer	7 (24.1)	22 (75.9)	2.28 (0.68–7.64)	0.181	0.79 (0.12–5.25)	0.803
Richest	4 (36.4)	7 (63.6)	4.10 (0.91–18.36)	0.066	1.03 (0.10–10.44)	0.982

ANC: antenatal care; AOR: adjusted odds ratio; CI: confidence interval; COR: crude odds ratio; km: kilometer; OR: odds ratio. Sample sizes vary due to missing responses; denominators are shown where applicable. – indicates variable not included in the multivariable model based on the pre-specified selection criterion (*p* < 0.20 in bivariate analysis) or not estimable due to sparse cells

In Xiengkhuang, religious affiliation was associated with MNH CoC completion. Compared with women identifying as Buddhist, those identifying with Tai Folk or other religions had lower odds of completing the MNH CoC (AOR 0.10; 95% CI 0.01–0.66). Other covariates included in the adjusted model were not statistically significant.

In Huaphanh, MNH CoC completion was associated with ethnicity and distance to the nearest health facility (Table 5). Minority ethnic group had lower odds of completing the MNH CoC than those identifying as Lao women (AOR: 0.17; 95% CI 0.03–0.96). In addition, compared with women living within 5 km of the nearest health facility, those living 6–10 km away (AOR: 0.16; 95% CI 0.03–0.94) and those living more than 10 km away (AOR: 0.13; 95% CI 0.02–0.83) had significantly lower odds of completing CoC.

### Factors associated with the completion of the MNCH CoC

To examine factors associated with the extended MNCH continuum of care (including childhood immunization) we fitted a pooled multivariate logistic regression model. Pooling was used due to the limited sample size of the immunization subsample. The extended MNCH CoC model demonstrated acceptable calibration (Hosmer–Lemeshow χ^2^(8) = 8.66, *p* = 0.278), with a pseudo-*R*^2^ of 0.166.

The timing of the first ANC visit was associated with completion of the extended MNCH CoC: women who initiated ANC at ≥ 3 months of pregnancy had lower odds of completing the full continuum compared with those initiated ANC at < 3 months (AOR: 0.27; 95% CI 0.10–0.70) (Table 6). In the pooled adjusted model, distance to the nearest health facility and ethnicity were not statistically associated with completion of the extended MNCH CoC.

**Table 6:** Factors associated with completion of the extended MNCH continuum of care (including childhood immunization), *n* = 152

Variables	Complete continuum of care		OR (95% CI)			
	Yes (%)	No (%)	COR	*p*	AOR	*p*
Age of women (years)						
15–24 years	7(11.7)	53(88.3)	1		1	
25–34 years	24(32.9)	49(67.1)	3.71 (1.47–9.40)	**0.006**	2.14 (0.79–5.76)	0.132
35–49 years	3 (15.8)	16 (84.2)	1.42 (0.33–6.16)	0.640	0.73 (0.15–3.67)	0.707
Number of family members						
≤ 3	2 (28.6)	5 (71.4)	1		–	
4–6	20 (25.3)	59 (74.7)	0.85 (0.15–4.74)	0.851	–	
≥ 7	12 (18.2)	54 (81.8)	0.56 (0.10–3.23)	0.513	–	
Education						
None, Preschool and primary	5 (9.1)	50 (90.9)	1			
Middle school	10 (25.0)	30 (75.0)	1.50(0.19–11.94)	0.702	–	
High school and above	19 (28.4)	48 (71.6)	3.79(0.68–21.21)	0.129	–	
Income generation						
Yes	28 (24.4)	87 (75.6)	1		1	
No	6 (14.6)	35 (85.4)	0.51 (0.19–1.34)	0.171	1.00 (0.29–3.43)	0.999
Religion						
Buddhism	26 (37.7)	43 (62.3)	1		1	
Tai Folk or others	8 (9.6)	75 (90.4)	0.18 (0.07–0.43)	< 0.001	0.19 (0.03–1.24)	0.083
Ethnicity						
Lao	26 (33.8)	51 (66.2)	1		1	
Minority	8 (10.7)	67 (89.3)	0.23 (0.10–0.56)	0.001	1.63 (0.25–10.51)	0.610
Road accessibility						
Yes	21 (25.0)	59 (73.8)	1		1	
Partially yes	9 (32.1)	19 (67.9)	1.33 (0.52–3.41)	0.551	1.28 (0.39–4.15)	0.683
No	4 (9.1)	40 (90.9)	0.28 (0.09–0.88)	0.030	0.77 (0.21–2.81)	0.695
Distance from the nearest health facility						
< 5 km	9 (28.1)	23 (71.9)	1		–	
6–10 km	9 (19.6)	37 (80.4)	0.62 (0.21–1.80)	0.381	–	
≥ 10 km	16 (21.6)	58 (78.4)	0.70 (0.27–1.83)	0.472	–	
No. of pregnancies						
1	9 (27.3)	24 (72.7)	1		–	
2	10 (20.0)	40 (80.0)	0.67 (0.24–1.88)	0.443	–	
≥ 3	15 (21.7)	54 (78.3)	0.74 (0.28–1.93)	0.540	–	
Timing of 1st ANC visit						
< 3 months^a^	23 (42.6)	31 (57.4)	1		1	
≥ 3 months	11 (11.2)	87 (88.8)	0.18 (0.08–0.43)	< 0.001	0.27 (0.10–0.70)	0.007
Informed about danger signs during pregnancy						
Yes	33 (23.4)	108 (76.6)	1		–	
No	1 (9.1)	10 (90.9)	0.33 (0.04–2.67)	0.297	–	
Wealth index (*n* = 148)						
Poorest	5 (17.2)	24 (82.8)	1		–	
Poorer	5 (16.7)	25 (83.3)	0.79 (0.20–3.07)	0.729	–	
Middle	9 (30.0)	21 (70.0)	2.06 (0.79–8.86)	0.116	–	
Richer	7 (23.3)	23 (76.7)	0.73 (0.15–3.51)	0.698	–	
Richest	8 (27.6)	21 (72.4)	1.52 (0.44–5.24)	0.505	–	

ANC: antenatal care; AOR: adjusted odds ratio; CI: confidence interval; COR: crude odds ratio; km: kilometer; OR: odds ratio. ^a^Participants reporting ‘don’t know’ for timing of first ANC were coded as ≥ 3 months

## Discussion

This study identified substantial gaps in the continuity of maternal, newborn, and child health care in two underserved provinces of Lao PDR. Overall, 36.2% of women completed the maternal and newborn health continuum of care, and completion fell to 22.4% when the continuum was extended to include six essential childhood vaccines, indicating that additional attrition after delivery as care transitions to postnatal and child health services.

Although these completion rates are higher than the 6.8% recorded previously in rural Khammouane using a modified composite coverage index [10], they remain lower than levels reported in Nepal (51%) [36], Cambodia (49%) [37], and Myanmar (43%) [38]. We used an "all-or-nothing" definition of completion to provide a clear and interpretable benchmark for program evaluation and to identify women who did not receive the full package of recommended contacts [39, 40]. While this approach does not capture partial service use, it is useful for locating points of attrition along the care pathway and for targeting populations at highest risk of gaps in care.

A consistent finding was the marked provincial disparity in completion. Xiengkhuang had higher completion than Huaphanh for both MNH CoC (49.7% vs 22.2%) and the extended MNCH CoC (28.9% vs 14.5%). This difference likely reflects variation in accessibility and underlying socioeconomic conditions; women in Xiengkhuang reported higher education levels, better road access, and closer proximity to health facilities than those in Huaphanh [12, 17]. Although both provinces have been supported by a bilateral health initiative since 2014, the persistently lower completion in Huaphanh suggests that structural barriers related to geography and health system capacity require more intensive and targeted interventions, such as mobile outreach teams and demand-side incentives for postnatal follow-up [15, 17, 18]. These findings highlight the need for context-specific strategies rather than uniform approaches applied across vulnerable areas [35, 36].

The factors associated with MNH CoC Completion also differed by province. In Xiengkhuang, religious affiliation (Tai Folk or other religions) was associated with lower odds of completion in the multivariate model. This may reflect differences in health-seeking behaviors and reliance on traditional practices or community-based decision-making that can reduce uptake of facility-based services [41–43]. This religion-related finding should be interpreted with caution. The Christian subgroup comprised only two participants, precluding meaningful comparison, and religious affiliation in this setting is closely intertwined with ethnicity, geography, and socioeconomic position; the observed association may, therefore, partly reflect these underlying factors rather than religion per se. In Huaphanh, MNH CoC completion was associated with ethnicity and distance to the nearest health facility (Table 5). This finding is consistent with evidence from Lao PDR that ethnic-minority women face compounded access barriers such as language discordance, different health beliefs and norms, lower health literacy, and weaker trust in and communication with healthcare providers [41, 44]. In addition, greater distance to the nearest health facility likely reduces CoC completion by increasing travel time and transport barriers, which in turn makes it harder for women to maintain repeated MNH contacts across the continuum [44, 45]. These province-specific patterns suggest that both structural and socio-cultural factors shape continuity, but the relative salience of these factors may differ across settings [17, 18].

Across the full lifecycle continuum, early engagement with antenatal care was associated with completion of the extended MNCH CoC. Women initiating ANC at ≥ 3 months (second/third trimester) had lower odds of completing the extended continuum than those initiating ANC within the first trimester, supporting the importance of early entry into care as an opportunity to establish follow-up scheduling, counseling, and linkage to subsequent services [9, 24, 46]. Programmatically, this emphasizes the value of strategies that encourage early pregnancy identification and timely initiation of ANC, including community outreach and demand-generation approaches appropriate to local contexts [11, 24, 47, 48].

Methodologically, we stratified the MNH CoC analysis by province to reflect distinct contextual environments but used a pooled multivariate model for the extended MNCH CoC, because the immunization subsample (*n* = 152) would yield unstable estimates if further stratified. We also assessed multicollinearity and found no evidence of problematic correlation among predictors (S1 Table). Sensitivity analysis indicated that the main findings were robust to assumptions about missing outcome-component data (S2 Table) and to alternative MNH CoC definitions with and without postpartum family planning (S3 Table).

The results have practical implications for improving continuity. In Xiengkhuang, engaging community and religious leaders—particularly within Tai Folk and other non-Buddhist communities—and incorporating culturally appropriate messaging about the benefits of antenatal, postnatal, and immunization care may help address socio-cultural barriers [20, 30, 47]. In Huaphanh, where completion rates were markedly lower and early ANC initiation was a significant barrier, targeted outreach to remote villages to support first-trimester ANC registration, together with active follow-up for postnatal and immunization contacts, may be particularly important. A pronounced gap also occurred within the postnatal period itself: postnatal-care coverage fell from 82.6% for mothers to 61.0% for newborns, a difference of 21.6 percentage points, identifying separate postnatal care for the newborn as one of the largest single points of attrition along the continuum and a key target for intervention. In both provinces, reducing attrition during the postnatal period remains critical; strengthening follow-up after delivery and improving linkage from facility-based delivery to postnatal and immunization services may help sustain continuity.

There are several limitations to this study. The cross-sectional design precludes causal inference, and self-reported service use may be subject to recall bias, particularly where documentation was unavailable. The wide confidence intervals for several regression estimates and the reduction from many significant bivariate associations to fewer significant associations in the multivariate models likely reflects the intercorrelation among socioeconomic predictors (e.g., education, wealth, road access) and the modest sample sizes, particularly in the province-stratified models. Furthermore, childhood immunization measurement deviated from standard definitions, because OPV3 was not consistently captured in the survey records, and OPV2 was used as a proxy, which may affect comparability with studies using the full schedule. However, applying the same definition across provinces supports internal comparison of geographic disparities. In addition, the purposive selection of districts with Type A hospitals limits generalizability to other districts or provinces that may have less developed health infrastructure. Accordingly, the wide confidence intervals around several adjusted odds ratios indicate limited precision and possible model instability in small subgroups, and these statistically significant associations should be interpreted with corresponding caution.

Finally, completion of recommended contacts does not necessarily indicate effective coverage, because service quality and content may vary. In addition, the mechanisms through which religion and ANC timing shape CoC completion—including health beliefs, social norms, decision-making authority, and provider interactions—could not be examined in this quantitative survey. Future research integrating household data with facility assessments and mixed-method approaches would help clarify how service quality and contextual factors shape both utilization and outcomes, and would support more targeted strategies to improve maternal, newborn, and child health in rural Lao PDR.

## Conclusion

Continuum completion remained low, particularly when extended to childhood immunization, and differed substantially between provinces. Determinants of completion varied by local context, underscoring the need for province-tailored strategies. Across the pooled extended continuum model, late ANC initiation was associated with lower completion, highlighting first-trimester entry as a key programmatic priority. Strengthening linkage from delivery to postnatal and child health services may help reduce drop-off across the lifecycle continuum. For Lao PDR, the priority actions that follow from these findings are to promote early, first-trimester antenatal care through community outreach and early pregnancy identification; to close the gap between facility delivery and postnatal care by scheduling postnatal and newborn contacts before discharge and reinforcing them through village health volunteers; and to tailor services to the populations least likely to complete the continuum—ethnic-minority women and those living far from facilities—through culturally appropriate, local-language counseling, engagement of community and religious leaders, and support for transport and referral. More broadly, other countries facing similar geographic, socioeconomic, and ethnic-diversity challenges are likely to benefit from the same principles: measuring continuity with a clear all-or-nothing indicator to expose where care breaks down, targeting the postnatal period as the most common point of attrition, and replacing uniform, one-size-fits-all programs with locally adapted, community-based strategies directed at the sub-populations with the lowest completion. Embedding these approaches within routine maternal and child health programs offers a practical route to strengthening continuity of care and, ultimately, reducing maternal, newborn, and child mortality in comparable low- and middle-income settings.

## Supplementary Information

Supplementary material 1. S1 Table. Multicollinearity diagnostics for multivariate regression models. This table presents the sub-category and individual variance inflation factors (VIF) for variables included in the adjusted MNH and MNCH models. Mean VIF values of 2.53 and 1.87 indicate no concerning multicollinearity among predictors.

Supplementary material 2. S2 Table. Sensitivity analysis for missing continuum-completion data. Factors associated with MNH CoC completion when conservative imputation was used for missing data, overall and by province.

Supplementary material 3. S3 Table. Sensitivity analysis using alternative MNH CoC definitions without postpartum family planning. Factors associated with MNH CoC completion when postpartum family planning is excluded from the all-or-nothing completion definition, overall and by province.

Supplementary material 4. S1 Figure. Retention and Completion Rates along the MNCH CoC. This figure illustrates the sequential completion and attrition of health services across the continuum for mothers and children in Xiengkhuang and Huaphanh provinces. The bars track the transition from ANC 4+ through SBA, PNCM/PNCN, and FP, which collectively define the MNH CoC completion rate at 36.2%. The final bar represents the extended MNCH CoC, which incorporates full childhood immunization, resulting in an overall completion rate of 22.4%. The data highlights a significant drop-off in service uptake as the continuum progresses, particularly during the transition to pediatric immunization services.

Supplementary material 5.

## Data Availability

The datasets used and analyzed during the current study is included in this manuscript and its supplementary information files.
