# Supplementary material for: Maternal, newborn, and child health continuum of care: completion and associated factors in Xiengkhuang and Huaphanh, Lao PDR
Source: Trop Med Health. 2026 Sep 4;54:189. doi: 10.1186/s41182-026-01066-7 (PMC13543367; doi:10.1186/s41182-026-01066-7)
Supplement: Supplementary file 1 [file 41182_2026_1066_MOESM1_ESM.docx]

**S1 Table. Multicollinearity diagnostics for multivariate regression models.**

| **Model** | **Variable Category** | **Sub-category** | **Individual VIF** | **Mean VIF** |
| --- | --- | --- | --- | --- |
| **MNH CoC Model** | **Age of women** | 24–34 years | 1.26 | **2.53** |
| (n=343) |  | 35–49 years | 1.23 |  |
|  | **Education** | Preschool & primary | 3.48 |  |
|  |  | Middle school | 4.20 |  |
|  |  | High school & above | 5.64 |  |
|  | **Religion** | Tai Folk or others | 4.63 |  |
|  | **Ethnicity** | Minority | 4.98 |  |
|  | **Road accessibility** | Partially yes | 1.32 |  |
|  |  | No | 1.75 |  |
|  | **Distance from Health Facility** | 6–10 km | 1.84 |  |
|  |  | More than 10 km | 2.13 |  |
|  | **Timing of 1st ANC** | 3 months ≤ | 1.13 |  |
|  | **Danger signs** | No | 1.14 |  |
|  | **Wealth Index** | Quintile 2–5 | 1.76–2.45 |  |
| **MNCH CoC Model** | **Age of women** | 24–34 years | 1.30 | **1.87** |
| (n=148) |  | 35–49 years | 1.23 |  |
|  | **Income Generation** | No | 1.15 |  |
|  | **Religion** | Tai Folk or others | 3.66 |  |
|  | **Ethnicity** | Minority | 3.72 |  |
|  | **Road accessibility** | Partially yes / No | 1.13–1.59 |  |
|  | **Timing of 1st ANC** | 3 months ≤ | 1.20 |  |
