# Supplementary material for: Maternal, newborn, and child health continuum of care: completion and associated factors in Xiengkhuang and Huaphanh, Lao PDR
Source: Trop Med Health. 2026 Sep 4;54:189. doi: 10.1186/s41182-026-01066-7 (PMC13543367; doi:10.1186/s41182-026-01066-7)
Supplement: Supplementary file 2 [file 41182_2026_1066_MOESM2_ESM.docx]

**S2 Table. Sensitivity analysis for missing continuum-completion data**

**A. Factors associated with MNH CoC completion when conservative imputation was used for missing data, overall (n=380)**

| MNH CoC |  | | OR (95% CI) | | | | | | |
| --- | --- | --- | --- | --- | --- | --- | --- | --- | --- |
|  | COR | | | *p* | | AOR (n=354) | | *p* | |
| **Age of women (years)** | |  |  | |  | |  | |  |
| 15–24 years | 1 | | |  | |  | |  | |
| 25–34 years | **2.26 (1.44-3.56)** | | | **<0.001** | | 1.70 (1.00-2.90) | | 0.052 | |
| 35–49 years | 1.40 (0.63-3.12) | | | 0.404 | | 0.81 (0.25-2.56) | | 0.717 | |
|  |  | | |  | |  | |  | |
| **Number of family members** | | | |  | |  | |  | |
| ≤3 | 1 | | |  | |  | |  | |
| 4-6 | 2.12 (0.66-6.82) | | | 0.207 | | - | | - | |
| ≥7 | 1.18 (0.36-3.87) | | | 0.779 | | - | | - | |
|  |  | | |  | |  | |  | |
| **Education** |  | | |  | |  | |  | |
| No education | 1 | | |  | | 1 | |  | |
| Preschool & primary | **8.53 (1.09-66.93)** | | | **0.041** | | 3.93 (0.43-36.03) | | 0.225 | |
| Middle school | **14.36 (1.87-110.41)** | | | **0.010** | | 6.25 (0.70-56.10) | | 0.102 | |
| High school & above | **21.38 (2.83-161.71)** | | | **0.003** | | 5.91 (0.68-51.12) | | 0.107 | |
|  |  | | |  | |  | |  | |
| **Income generation** |  | | |  | |  | |  | |
| Yes | 1 | | |  | |  | |  | |
| No | 0.76 (0.47-1.22) | | | 0.253 | | - | | - | |
|  |  | | |  | |  | |  | |
| **Religion** |  | | |  | |  | |  | |
| Buddhism | 1 | | |  | | 1 | |  | |
| Tai Folk or others | **0.25 (0.16-0.39)** | | | **<0.001** | | **0.26 (0.09-0.79)** | | **0.017** | |
|  |  | | |  | |  | |  | |
| **Ethnicity** |  | | |  | |  | |  | |
| Lao | 1 | | |  | | 1 | |  | |
| Minority | **0.31 (0.20-0.48)** | | | **<0.001** | | 1.45 (0.46-4.58) | | 0.524 | |
|  |  | | |  | |  | |  | |
| **Road accessibility** |  | | |  | |  | |  | |
| Yes | 1 | | |  | | 1 | |  | |
| Partially yes | 1.35 (0.80-2.26) | | | 0.260 | | 1.61 (0.85-3.06) | | 0.144 | |
| No | **0.24 (0.13-0.44)** | | | **<0.001** | | 0.71 (0.35-1.46) | | 0.355 | |
|  |  | | |  | |  | |  | |
| **Distance from the nearest health facility** | | | |  | |  | |  | |
| <5 km | 1 | | |  | | 1 | |  | |
| 6–10 km | 0.81 (0.47-1.42) | | | 0.470 | | 0.69 (0.35-1.37) | | 0.290 | |
| >10 km | **0.42 (0.24-0.74)** | | | **0.003** | | 0.72 (0.36-1.42) | | 0.340 | |
|  |  | | |  | |  | |  | |
| **No. of pregnancies** |  | | |  | |  | |  | |
| 1 | 1 | | |  | |  | |  | |
| 2 | 1.12 (0.65-1.91) | | | 0.692 | | - | |  | |
| ≥3 | 0.85 (0.51-1.42) | | | 0.531 | | - | |  | |
|  |  | | |  | |  | |  | |
| **Timing of 1^st^ ANC visit** | (n=363) | | |  | |  | |  | |
| <3 months | 1 | | |  | | 1 | |  | |
| ≥3 months | **0.44 (0.28-0.69)** | | | **<0.001** | | 0.62 (0.36-1.06) | | 0.082 | |
|  |  | | |  | |  | |  | |
| **Informed about danger signs during pregnancy** | | | | | |  | |  | |
| Yes | 1 | | |  | | 1 | |  | |
| No | **0.30 (0.13-0.68)** | | | **0.004** | | 0.37 (0.13-1.00) | | 0.051 | |
|  |  | | |  | |  | |  | |
| **Wealth index** | (n=371) | | |  | |  | |  | |
| Poorest | 1 | | |  | | 1 | |  | |
| Poorer | 1.46 (0.69-3.09) | | | 0.322 | | 0.84 (0.36-1.97) | | 0.684 | |
| Middle | **2.12 (1.02-4.39)** | | | **0.043** | | 1.18 (0.51-2.73) | | 0.702 | |
| Richer | **2.24 (1.09-4.64)** | | | **0.029** | | 1.02 (0.42-2.46) | | 0.965 | |
| Richest | **4.58 (2.23-9.40)** | | | **<0.001** | | 1.36 (0.55-3.37) | | 0.507 | |

Child Immunization: one dose of the Bacillus Calmette–Guérin, two doses of Oral Polio Vaccine, one dose of Inactivated Polio Vaccine, three doses of Pentavalent vaccine, three doses of Pneumococcal Conjugate vaccine and one dose of Measles-Rubella vaccine.

ANC, antenatal care; AOR, adjusted odds ratio; CI, confidence interval; COR, crude odds ratio; km, kilometer; OR, odds ratio

**B. Factors associated with MNH CoC completion when conservative imputation was used for missing data, Xiengkhuang (n=190).**

| MNH CoC |  | | OR (95% CI) | | | | | | |
| --- | --- | --- | --- | --- | --- | --- | --- | --- | --- |
|  | COR | | | *p* | | AOR(n=190) | | *p* | |
| **Age of women (years)** | |  |  | |  | |  | |  |
| 15–24 years | 1 | | |  | | 1 | |  | |
| 25–34 years | **2.03 (1.09-3.77)** | | | **0.026** | | 1.62 (0.83-3.13) | | 0.155 | |
| 35–49 years | 0.91 (0.34-2.48) | | | 0.858 | | 0.66 (0.19-2.30) | | 0.516 | |
|  |  | | |  | |  | |  | |
| **Number of family members** | | | |  | |  | |  | |
| ≤3 | 1 | | |  | |  | |  | |
| 4-6 | 2.59 (0.45-14.86) | | | 0.285 | | - | | - | |
| ≥7 | 1.39 (0.24-8.05) | | | 0.715 | | - | | - | |
|  |  | | |  | |  | |  | |
| **Education** |  | | |  | |  | |  | |
| No education | 1 | | |  | |  | |  | |
| Preschool & primary | 1.62 (0.15-17.35) | | | 0.688 | | - | | - | |
| Middle school | 2.88 (028-29.80) | | | 0.374 | | - | | - | |
| High school & above | 3.68 (0.37-36.87) | | | 0.268 | | - | | - | |
|  |  | | |  | |  | |  | |
| **Income generation** |  | | |  | |  | |  | |
| Yes | 1 | | |  | | 1 | |  | |
| No | **0.47 (0.26-0.86)** | | | **0.014** | | 0.67 (0.34-1.29) | | 0.220 | |
|  |  | | |  | |  | |  | |
| **Religion** |  | | |  | |  | |  | |
| Buddhism | 1 | | |  | | 1 | |  | |
| Tai Folk or others | **0.38 (0.21-0.68)** | | | **0.001** | | **0.10 (0.01-0.67)** | | **0.018** | |
|  |  | | |  | |  | |  | |
| **Ethnicity** |  | | |  | |  | |  | |
| Lao | 1 | | |  | | 1 | |  | |
| Minority | **0.49 (0.27-0.88)** | | | **0.017** | | 6.68 (0.94-47.33) | | 0.057 | |
|  |  | | |  | |  | |  | |
| **Road accessibility** |  | | |  | |  | |  | |
| Yes | 1 | | |  | | 1 | |  | |
| Partially yes | 0.84 (0.43-1.67) | | | 0.626 | | 0.95 (0.44-2.04) | | 0.896 | |
| No | **0.31 (0.14-0.71)** | | | **0.006** | | 0.40 (0.16-1.00) | | 0.051 | |
|  |  | | |  | |  | |  | |
| **Distance from the nearest health facility** | | | |  | |  | |  | |
| <5 km | 1 | | |  | |  | |  | |
| 6–10 km | 1.28 (0.64-2.56) | | | 0.483 | | - | | - | |
| >10 km | 1.00 (0.49-2.05) | | | 1.000 | | - | | - | |
|  |  | | |  | |  | |  | |
| **No. of pregnancies** |  | | |  | |  | |  | |
| 1 | 1 | | |  | |  | |  | |
| 2 | 1.26 (0.59-2.70) | | | 0.543 | | - | |  | |
| ≥3 | 0.91 (0.47-1.76) | | | 0.781 | | - | |  | |
|  |  | | |  | |  | |  | |
| **Timing of 1^st^ ANC visit** |  | | |  | |  | |  | |
| <3 months | 1 | | |  | | 1 | |  | |
| ≥3 months | **0.50 (0.28-0.91)** | | | **0.022** | | 0.64 (0.33-1.23) | | 0.181 | |
|  |  | | |  | |  | |  | |
| **Informed about danger signs during pregnancy** | | | | | |  | |  | |
| Yes | 1 | | |  | |  | |  | |
| No | 1.41 (0.31-6.50) | | | 0.661 | | - | | - | |
|  |  | | |  | |  | |  | |
| **Wealth index** |  | | |  | |  | |  | |
| Poorest | 1 | | |  | |  | |  | |
| Poorer | 0.38 (0.12-1.27) | | | 0.115 | | - | | - | |
| Middle | 1.44 (0.49-4.27) | | | 0.511 | | - | | - | |
| Richer | 1.15 (0.41-3.22) | | | 0.797 | | - | | - | |
| Richest | 1.71 (0.64-4.55) | | | 0.285 | | - | | - | |

ANC, antenatal care; AOR, adjusted odds ratio; CI, confidence interval; COR, crude odds ratio; km, kilometer; OR, odds ratio

**C. Factors associated with MNH CoC completion when conservative imputation was used for missing data, Huaphanh (n=190)**

| MNH CoC |  | | OR (95% CI) | | | | | | |
| --- | --- | --- | --- | --- | --- | --- | --- | --- | --- |
|  | COR | | | *p* | | AOR (n=164) | | *p* | |
| **Age of women (years)** | |  |  | |  | |  | |  |
| 15–24 years | 1 | | |  | |  | |  | |
| 25–34 years | 1.81 (0.87-3.76) | | | 0.113 | | - | |  | |
| 35–49 years | 1.65 (0.40-6-72) | | | 0.486 | | - | |  | |
|  |  | | |  | |  | |  | |
| **Number of family members** | | | |  | |  | |  | |
| ≤3 | 1 | | |  | |  | |  | |
| 4-6 | 1.39 (0.28-7.01) | | | 0.691 | | - | | - | |
| ≥7 | 0.74 (0.14-3.92) | | | 0.726 | | - | | - | |
|  |  | | |  | |  | |  | |
| **Education** |  | | |  | |  | |  | |
| None, Preschool & primary | 1 | | |  | | 1 | |  | |
| Middle school | 2.30 (0.87-6.14) | | | 0.095 | | 1.26 (0.28-5.73) | | 0.767 | |
| High school & above | **3.42 (1.38-8.47)** | | | **0.008** | | 0.99 (0.23-4.27) | | 0.993 | |
|  |  | | |  | |  | |  | |
| **Income generation** |  | | |  | |  | |  | |
| Yes | 1 | | |  | | 1 | |  | |
| No | **0.25 (0.12-0.52)** | | | **<0.001** | | 0.55 (0.11-2.67) | | 0.457 | |
|  |  | | |  | |  | |  | |
| **Religion** |  | | |  | |  | |  | |
| Buddhism | 1 | | |  | | 1 | |  | |
| Tai Folk or others | **0.27 (0.13-0.56)** | | | **<0.001** | | 1.15 (0.22-5.86) | | 0.869 | |
|  |  | | |  | |  | |  | |
| **Ethnicity** |  | | |  | |  | |  | |
| Lao | 1 | | |  | | 1 | |  | |
| Minority | **0.49 (0.27-0.88)** | | | **0.017** | | **0.07 (0.01-0.70)** | | **0.024** | |
|  |  | | |  | |  | |  | |
| **Road accessibility** |  | | |  | |  | |  | |
| Yes | 1 | | |  | | 1 | |  | |
| Partially yes | **2.61 (1.12-6.06)** | | | **0.026** | | 2.81 (0.60-13.22) | | 0.191 | |
| No | **0.25 (0.09-0.72)** | | | **0.010** | | 2.35 (0.35-15.62) | | 0.377 | |
|  |  | | |  | |  | |  | |
| **Distance from the nearest health facility** | | | |  | |  | |  | |
| <5 km | 1 | | |  | | 1 | |  | |
| 6–10 km | 0.56 (0.20-1.58) | | | 0.273 | | **0.07 (0.01-0.92)** | | **0.043** | |
| >10 km | **0.22 (0.08-0.65)** | | | **0.006** | | 0.10 (0.01-1.18) | | 0.068 | |
|  |  | | |  | |  | |  | |
| **No. of pregnancies** |  | | |  | |  | |  | |
| 1 | 1 | | |  | |  | |  | |
| 2 | 1.81 (0.71-4.57) | | | 0.213 | | - | |  | |
| ≥3 | 1.00 (0.38-2.63) | | | 0.993 | | - | |  | |
|  |  | | |  | |  | |  | |
| **Timing of 1^st^ ANC visit** | (n=173) | | |  | |  | |  | |
| <3 months | 1 | | |  | | 1 | |  | |
| ≥3 months | **0.43 (0.19-0.96)** | | | **0.040** | | 0.44 (0.16-1.27) | | 0.130 | |
|  |  | | |  | |  | |  | |
| **Informed about danger signs during pregnancy** | | | | | |  | |  | |
| Yes | 1 | | |  | | 1 | |  | |
| No | **0.26 (0.07-0.89)** | | | **0.031** | | **0.06 (0.01-0.38)** | | **0.003** | |
|  |  | | |  | |  | |  | |
| **Wealth index** | (n=181) | | |  | |  | |  | |
| Poorest | 1 | | |  | | 1 | |  | |
| Poorer | **3.54 (1.22-10.22)** | | | **0.020** | | 2.17(0.44-10.73) | | 0.343 | |
| Middle | 2.20 (0.71-6.82) | | | 0.171 | | 1.20 (017-8.58) | | 0.854 | |
| Richer | 2.28 (0.69-7.58) | | | 0.177 | | 0.81 (0.13-5.15) | | 0.825 | |
| Richest | 4.48 (1.00-20.02) | | | 0.050 | | 1.64 (0.13-20.07) | | 0.697 | |

ANC, antenatal care; AOR, adjusted odds ratio; CI, confidence interval; COR, crude odds ratio; km, kilometer; OR, odds ratio

**D. Factors associated with extended MNCH CoC completion when complete case analysis was used for missing data (n=148)**

| MNCH CoC |  | | OR (95% CI) | | | | | | |
| --- | --- | --- | --- | --- | --- | --- | --- | --- | --- |
|  | COR | | | *p* | | AOR (n=148) | | *p* | |
| **Age of women (years)** | |  |  | |  | |  | |  |
| 15–24 years | 1 | | |  | |  | |  | |
| 25–34 years | **3.71 (1.46-9.40)** | | | **0.006** | | 2.11 (0.79-5.67) | | 0.138 | |
| 35–49 years | 1.42 (0.33-6.16) | | | 0.640 | | 0.70 (0.15-3.25) | | 0.651 | |
|  |  | | |  | |  | |  | |
| **Number of family members** | | | |  | |  | |  | |
| ≤3 | 1 | | |  | |  | |  | |
| 4-6 | 0.85 (0.15-4.74) | | | 0.851 | | - | | - | |
| ≥7 | 0.56 (0.10-3.23) | | | 0.513 | | - | | - | |
|  |  | | |  | |  | |  | |
| **Education** |  | | |  | |  | |  | |
| None, Preschool & primary | 1 | | |  | | 1 | |  | |
| Middle school | 2.67 (0.82-8.65) | | | 0.102 | | 2.24 (0.65-9.04) | | 0.190 | |
| High school & above | **3.17 (1.08-9.27)** | | | **0.035** | | 1.47 (0.36-6.00) | | 0.591 | |
|  |  | | |  | |  | |  | |
| **Income generation** |  | | |  | |  | |  | |
| Yes | 1 | | |  | |  | |  | |
| No | 0.51 (0.19-1.34) | | | 0.171 | | - | | - | |
|  |  | | |  | |  | |  | |
| **Religion** |  | | |  | |  | |  | |
| Buddhism | 1 | | |  | | 1 | |  | |
| Tai Folk or others | **0.18 (0.07-0.43)** | | | **<0.001** | | 0.17 (0.02-1.75) | | 0.137 | |
|  |  | | |  | |  | |  | |
| **Ethnicity** |  | | |  | |  | |  | |
| Lao | 1 | | |  | | 1 | |  | |
| Minority | **0.23 (0.10-0.56)** | | | **0.001** | | 1.52 (0.14-16.48) | | 0.731 | |
|  |  | | |  | |  | |  | |
| **Road accessibility** |  | | |  | |  | |  | |
| Yes | 1 | | |  | | 1 | |  | |
| Partially yes | 1.33 (0.52-3.41) | | | 0.551 | | 1.31 (0.40-4.34) | | 0.657 | |
| No | **0.28 (0.09-0.88)** | | | **0.030** | | 0.93 (0.25-3.45) | | 0.910 | |
|  |  | | |  | |  | |  | |
| **Distance from the nearest health facility** | | | |  | |  | |  | |
| <5 km | 1 | | |  | |  | |  | |
| 6–10 km | 0.62 (0.21-1.80) | | | 0.381 | | - | |  | |
| >10 km | 0.70 (0.27-1.83) | | | 0.472 | | - | |  | |
|  |  | | |  | |  | |  | |
| **No. of pregnancies** |  | | |  | |  | |  | |
| 1 | 1 | | |  | |  | |  | |
| 2 | 0.67 (0.24-1.88) | | | 0.443 | | - | |  | |
| ≥3 | 0.74 (0.28-1.93) | | | 0.540 | | - | |  | |
|  |  | | |  | |  | |  | |
| **Timing of 1^st^ ANC visit** | (n=148) | | |  | |  | |  | |
| <3 months | 1 | | |  | | 1 | |  | |
| ≥3 months | **0.25 (0.11-0.56)** | | | **0.001** | | **0.38 (0.16-0.92)** | | **0.033** | |
|  |  | | |  | |  | |  | |
| **Informed about danger signs during pregnancy** | | | | | |  | |  | |
| Yes | 1 | | |  | |  | |  | |
| No | 0.33 (0.04-2.67) | | | 0.297 | | - | |  | |
|  |  | | |  | |  | |  | |
| **Wealth index** | (n=148) | | |  | |  | |  | |
| Poorest | 1 | | |  | |  | |  | |
| Poorer | 0.79 (0.20-3.07) | | | 0.729 | | - | |  | |
| Middle | 2.64 (0.79-8.86) | | | 0.116 | | - | |  | |
| Richer | 0.73 (0.15-3.51) | | | 0.698 | | - | |  | |
| Richest | 1.52 (0.44-5.24) | | | 0.505 | | - | |  | |

ANC, antenatal care; AOR, adjusted odds ratio; CI, confidence interval; COR, crude odds ratio; km, kilometer; OR, odds ratio
