# Supplementary material for: Maternal, newborn, and child health continuum of care: completion and associated factors in Xiengkhuang and Huaphanh, Lao PDR
Source: Trop Med Health. 2026 Sep 4;54:189. doi: 10.1186/s41182-026-01066-7 (PMC13543367; doi:10.1186/s41182-026-01066-7)
Supplement: Supplementary file 3 [file 41182_2026_1066_MOESM3_ESM.docx]

**S3 Table. Sensitivity analysis using alternative MNH CoC definitions without postpartum family planning.**  **A. Factor associated with completing MNH CoC without FP in the survey area (n=373)**

| MNH CoC | OR (95% CI) | | | | | | | | |
| --- | --- | --- | --- | --- | --- | --- | --- | --- | --- |
|  | COR | | | *p* | | AOR (n=348) | | *p* | |
| **Age of women (years)** | |  |  | |  | |  | |  |
| 15–24 years | 1 | | |  | | 1 | |  | |
| 25–34 years | **1.80 (1.17-2.76)** | | | **0.007** | | 1.23 (0.72-2.10) | | 0.453 | |
| 35–49 years | 2.23 (1.02-4.89) | | | 0.044 | | 1.46 (0.42-5.14) | | 0.553 | |
|  |  | | |  | |  | |  | |
| **Number of family members** | | | |  | |  | |  | |
| ≤3 | 1 | | |  | |  | |  | |
| 4-6 | 1.49 (0.50-4.41) | | | 0.475 | | - | | - | |
| ≥7 | 0.60 (0.20-1.80) | | | 0.364 | | - | | - | |
|  |  | | |  | |  | |  | |
| **Education** |  | | |  | |  | |  | |
| No education | 1 | | |  | | 1 | |  | |
| Preschool & primary | **7.45 (1.65-33.71)** | | | **0.009** | | 2.76 (0.63-12.15) | | 0.178 | |
| Middle school | **13.00 (2.92-57.89)** | | | **0.001** | | 4.09 (0.97-17.23) | | 0.055 | |
| High school & above | **20.49 (4.68-89.75)** | | | **0.000** | | 4.01 (0.95-16.98) | | 0.060 | |
|  |  | | |  | |  | |  | |
| **Income generation** |  | | |  | |  | |  | |
| Yes | 1 | | |  | |  | |  | |
| No | 0.73 (0.47-1.14) | | | 0.169 | | - | | - | |
|  |  | | |  | |  | |  | |
| **Religion** |  | | |  | |  | |  | |
| Buddhism | 1 | | |  | | 1 | |  | |
| Tai Folk or others | **0.14 (0.09-0.23)** | | | **<0.001** | | **0.28 (0.10-0.84)** | | **0.023** | |
|  |  | | |  | |  | |  | |
| **Ethnicity** |  | | |  | |  | |  | |
| Lao | 1 | | |  | | 1 | |  | |
| Minority | **0.17 (0.1-0.26)** | | | **<0.001** | | 0.75 (0.25-2.24) | | 0.600 | |
|  |  | | |  | |  | |  | |
| **Road accessibility** |  | | |  | |  | |  | |
| Yes | 1 | | |  | | 1 | |  | |
| Partially yes | 1.20 (0.71-2.05) | | | 0.496 | | 1.34 (0.70-2.58) | | 0.374 | |
| No | **0.24 (0.14-0.41)** | | | **<0.001** | | 0.84 (0.43-1.65) | | 0.612 | |
|  |  | | |  | |  | |  | |
| **Distance from the nearest health facility** | | | |  | |  | |  | |
| <5 km | 1 | | |  | | 1 | |  | |
| 6–10 km | 1.18 (0.67-2.07) | | | 0.564 | | 1.09 (0.54-2.21) | | 0.805 | |
| >10 km | **0.44 (0.26-0.77)** | | | **0.004** | | 0.62 (0.30-1.30) | | 0.207 | |
|  |  | | |  | |  | |  | |
| **No. of pregnancies** |  | | |  | |  | |  | |
| 1 | 1 | | |  | |  | |  | |
| 2 | 0.75 (0.44-1.27) | | | 0.278 | | - | |  | |
| ≥3 | 0.66 (0.40-1.09) | | | 0.103 | | - | |  | |
|  |  | | |  | |  | |  | |
| **Timing of 1^st^ ANC visit** |  | | |  | |  | |  | |
| <3 months | 1 | | |  | | 1 | |  | |
| ≥3 months | **0.51 (0.33-0.79)** | | | **0.003** | | 0.76 (0.44-1.33) | | 0.343 | |
|  |  | | |  | |  | |  | |
| **Informed about danger signs during pregnancy** | | | | | |  | |  | |
| Yes | 1 | | |  | | 1 | |  | |
| No | **0.23 (0.11-0.50)** | | | **<0.001** | | **0.30 (0.11-0.82)** | | **0.019** | |
|  |  | | |  | |  | |  | |
| **Wealth index** |  | | |  | |  | |  | |
| Poorest | 1 | | |  | | 1 | |  | |
| Poorer | 1.48 (0.74-2.97) | | | 0.268 | | 0.94 (0.40-2.16) | | 0.875 | |
| Middle | **2.52 (1.26-5.01)** | | | **0.009** | | 1.49 (0.66-3.37) | | 0.339 | |
| Richer | **2.65 (1.34-5.26)** | | | **0.005** | | 0.99 (0.43-2.27) | | 0.978 | |
| Richest | **8.63 (4.06-18.33)** | | | **<0.001** | | 2.01 (0.79-5.09) | | 0.141 | |

**B. Factors associated with completing MNH CoC without FP in Xiengkouang (n=190)**

| MNH CoC | OR (95% CI) | | | | | | | | |  |
| --- | --- | --- | --- | --- | --- | --- | --- | --- | --- | --- |
|  | COR | | | *p* | | AOR (n=190) | | *p* | |  |
| **Age of women (years)** | |  |  | |  | |  | |  |  |
| 15–24 years | 1 | | |  | | 1 | |  | |  |
| 25–34 years | 1.84 (0.96-3.54) | | | 0.067 | | - | |  | |  |
| 35–49 years | 1.69 (0.58-4.87) | | | 0.334 | | - | |  | |  |
|  |  | | |  | |  | |  | |  |
| **Number of family members** | | | |  | |  | |  | |  |
| ≤3 | 1 | | |  | | - | |  | |  |
| 4-6 | 1.80 (0.31-10.50) | | | 0.516 | | - | |  | |  |
| ≥7 | 0.62 (0.11-3.60) | | | 0.596 | | - | |  | |  |
|  |  | | |  | |  | |  | |  |
| **Education** |  | | |  | |  | |  | |  |
| No education | 1 | | |  | | - | |  | |  |
| Preschool & primary | 1.18 (0.15-9.32) | | | 0.878 | | - | |  | |  |
| Middle school | 1.83 (0.24-14.221) | | | 0.562 | | - | |  | |  |
| High school & above | 3.08 (0.41-2.21) | | | 0.274 | | - | |  | |  |
|  |  | | |  | |  | |  | |  |
| **Income generation** |  | | |  | |  | |  | |  |
| Yes | 1 | | |  | | 1 | |  | |  |
| No | **0.31 (0.16-0.58)** | | | **<0.001** | | **0.38 (0.17-0.85)** | | **0.019** | |  |
|  |  | | |  | |  | |  | |  |
| **Religion** |  | | |  | |  | |  | |  |
| Buddhism | 1 | | |  | | 1 | |  | |  |
| Tai Folk or others | **0.14 (0.07-0.28)** | | | **<0.001** | | **0.07 (0.01-0.44)** | | **0.005** | |  |
|  |  | | |  | |  | |  | |  |
| **Ethnicity** |  | | |  | |  | |  | |  |
| Lao | 1 | | |  | | 1 | |  | |  |
| Minority | **0.20 (0.10-3.79)** | | | **<0.001** | | 3.08 (0.49-19.46) | | 0.231 | |  |
|  |  | | |  | |  | |  | |  |
| **Road accessibility** |  | | |  | |  | |  | |  |
| Yes | 1 | | |  | | 1 | |  | |  |
| Partially yes | 0.82 (0.39-1.74) | | | 0.612 | | 1.41 (0.51-3.88) | | 0.504 | |  |
| No | **0.36 (0.17-0.80)** | | | **0.012** | | 0.73 (0.26-2.06) | | 0.551 | |  |
|  |  | | |  | |  | |  | |  |
| **Distance from the nearest health facility** | | | |  | |  | |  | |  |
| <5 km | 1 | | |  | | 1 | |  | |  |
| 6–10 km | **2.62 (1.21-5.65)** | | | **0.014** | | **2.77 (1.11-6.89)** | | **0.028** | |  |
| >10 km | 1.33 (0.63-2.78) | | | 0.454 | | 2.05 (0.82-5.14) | | 0.124 | |  |
|  |  | | |  | |  | |  | |  |
| **No. of pregnancies** |  | | |  | |  | |  | |  |
| 1 | 1 | | |  | | 1 | |  | |  |
| 2 | 0.92 (0.40-2.11) | | | 0.851 | | - | |  | |  |
| ≥3 | 0.72 (0.36-1.46) | | | 0.367 | | - | |  | |  |
|  |  | | |  | |  | |  | |  |
| **Timing of 1^st^ ANC visit** |  | | |  | |  | |  | |  |
| <3 months | 1 | | |  | | 1 | |  | |  |
| ≥3 months | 0.60 (0.32-1.13) | | | 0.114 | | - | |  | |  |
|  |  | | |  | |  | |  | |  |
| **Informed about danger signs during pregnancy** | | | | | |  | |  | |  |
| Yes | 1 | | |  | | 1 | |  | |  |
| No | 1.19 (0.22-6.34) | | | 0.839 | | **-** | |  | |  |
|  |  | | |  | |  | |  | |  |
| **Wealth index** |  | | |  | |  | |  | |  |
| Poorest | 1 | | |  | | 1 | |  | |  |
| Poorer | 0.65 (0.21-1.98) | | | 0.446 | | 0.71 (0.19-2.69) | | 0.616 | |  |
| Middle | 1.38 (0.45-4.24) | | | 0.569 | | 0.66 (0.18-2.38) | | 0.526 | |  |
| Richer | 1.43 (0.49-4.16) | | | 0.507 | | 0.80 (0.22-2.95) | | 0.735 | |  |
| Richest | **2.94 (1.02-8.49)** | | | **0.046** | | 0.80 (0.21-3.06) | | 0.748 | |  |

**C. Factor associated with completing MNH CoC without FP in Huaphanh (n=183)**

| MNH CoC | | OR (95% CI) | | | | | | | | |
| --- | --- | --- | --- | --- | --- | --- | --- | --- | --- | --- |
|  |  | COR | | | *p* | | AOR (n=174) | | *p* | |
| **Age of women (years)** | | |  |  | |  | |  | |  |
| 15–24 years | | 1 | | |  | | 1 | |  | |
| 25–34 years | | 1.15 (0.60-2.24) | | | 0.671 | | - | |  | |
| 35–49 years | | 1.64 (0.43-6.27) | | | 0.466 | | - | |  | |
|  | |  | | |  | |  | |  | |
| **Number of family members** | | | | |  | |  | |  | |
| ≤3 | | 1 | | |  | | - | |  | |
| 4-6 | | 1.10 (0.25-4.91) | | | 0.899 | | - | |  | |
| ≥7 | | 0.40 (0.09-1.88) | | | 0.248 | | - | |  | |
|  | |  | | |  | |  | |  | |
| **Education** | |  | | |  | |  | |  | |
| None, Preschool & primary | | 1 | | |  | | 1 | |  | |
| Middle school | | **3.13 (1.32-7.41)** | | | **0.009** | | 2.15 (0.71-6.49) | | 0.174 | |
| High school & above | | **3.71 (1.63-8.40)** | | | **0.002** | | 1.26 (0.41-3.87) | | 0.681 | |
|  | |  | | |  | |  | |  | |
| **Income generation** | |  | | |  | |  | |  | |
| Yes | | 1 | | |  | | - | |  | |
| No | | 1.01 (0.47-2.18) | | | 0.980 | | **-** | |  | |
|  | |  | | |  | |  | |  | |
| **Religion** | |  | | |  | |  | |  | |
| Buddhism | | 1 | | |  | | 1 | |  | |
| Tai Folk or others | | **0.14 (0.07-0.28)** | | | **<0.001** | | 0.51 (0.09-2.81) | | 0.439 | |
|  | |  | | |  | |  | |  | |
| **Ethnicity** | |  | | |  | |  | |  | |
| Lao | | 1 | | |  | | 1 | |  | |
| Minority | | **0.21 (0.11-0.42)** | | | **<0.001** | | 0.23 (0.05-1.15) | | 0.073 | |
|  | |  | | |  | |  | |  | |
| **Road accessibility** | |  | | |  | |  | |  | |
| Yes | | 1 | | |  | | 1 | |  | |
| Partially yes | | 1.75 (0.77-3.96) | | | 0.181 | | - | |  | |
| No | |  | | |  | |  | |  | |
|  | |  | | |  | |  | |  | |
| **Distance from the nearest health facility** | | | | |  | |  | |  | |
| <5 km | | 1 | | |  | | 1 | |  | |
| 6–10 km | | 0.73 (0.26-2.06) | | | 0.546 | | 0.46 (0.12-1.83) | | 0.269 | |
| >10 km | | **0.23 (0.08-0.67)** | | | **0.007** | | **0.11 (0.02-0.53)** | | **0.006** | |
|  | |  | | |  | |  | |  | |
| **No. of pregnancies** | |  | | |  | |  | |  | |
| 1 | | 1 | | |  | |  | |  | |
| 2 | | 1.08 (0.48-2.45) | | | 0.848 | | - | |  | |
| ≥3 | | 0.73 (0.32-1.67) | | | 0.453 | | - | |  | |
|  | |  | | |  | |  | |  | |
| **Timing of 1^st^ ANC visit** | |  | | |  | |  | |  | |
| <3 months | | 1 | | |  | |  | |  | |
| ≥3 months | | 0.54 (0.26-1.10) | | | 0.090 | | - | |  | |
|  | |  | | |  | |  | |  | |
| **Informed about danger signs during pregnancy** | | | | | | |  | |  | |
| Yes | | 1 | | |  | | 1 | |  | |
| No | | **0.23 (0.08-0.68)** | | | **0.008** | | **0.13 (0.03-0.62)** | | **0.011** | |
|  | |  | | |  | |  | |  | |
| **Wealth index** | |  | | |  | |  | |  | |
| Poorest | | 1 | | |  | | 1 | |  | |
| Poorer | | 2.39 (0.89-6.44) | | | 0.085 | | 2.32 (0.78-6.90) | | 0.129 | |
| Middle | | **3.20 (1.18-8.69)** | | | **0.022** | | 2.16 (0.59-7.90) | | 0.243 | |
| Richer | | 2.44 (0.84-7.12) | | | 0.103 | | 0.68 (0.18-2.56) | | 0.5676 | |
| Richest | | **8.97 (2.11-38.14)** | | | **0.003** | | 1.73 (0.28-10.80) | | 0.556 | |
|  |  |  |  |  |  |  |  |  |  |  |
